# Convergent roles of de novo mutations and common variants in schizophrenia in tissue-specific and spatiotemporal co-expression network

**DOI:** 10.1038/s41398-018-0154-2

**Published:** 2018-05-24

**Authors:** Peilin Jia, Xiangning Chen, Ayman H. Fanous, Zhongming Zhao

**Affiliations:** 1 Center for Precision Health, School of Biomedical Informatics, The University of Texas Health Science Center at Houston, Houston, TX USA; 2 Nevada Institute of Personalized Medicine and Department of Psychology, University of Nevada Las Vegas, Las Vegas, NV USA; 3 Virginia Institute for Psychiatric and Behavioral Genetics, Virginia Commonwealth University, Richmond, VA USA; 4 Department of Psychiatry, Keck School of Medicine of the University of Southern California, Los Angeles, CA USA; 5 Mental Health Service Line, Washington VA Medical Center, Washington, DC USA; 6 Department of Psychiatry, Georgetown University School of Medicine, Washington, DC USA; 7 Human Genetics Center, School of Public Health, The University of Texas Health Science Center at Houston, Houston, TX USA; 8 Department of Biomedical Informatics, Vanderbilt University Medical Center, Nashville, TN USA

## Abstract

Genetic components susceptible to complex disease such as schizophrenia include a wide spectrum of variants, including common variants (CVs) and de novo mutations (DNMs). Although CVs and DNMs differ by origin, it remains elusive whether and how they interact at the gene, pathway, and network levels that leads to the disease. In this work, we characterized the genes harboring schizophrenia-associated CVs (CVgenes) and the genes harboring DNMs (DNMgenes) using measures from network, tissue-specific expression profile, and spatiotemporal brain expression profile. We developed an algorithm to link the DNMgenes and CVgenes in spatiotemporal brain co-expression networks. DNMgenes tended to have central roles in the human protein–protein interaction (PPI) network, evidenced in their high degree and high betweenness values. DNMgenes and CVgenes connected with each other significantly more often than with other genes in the networks. However, only CVgenes remained significantly connected after adjusting for their degree. In our gene co-expression PPI network, we found DNMgenes and CVgenes connected in a tissue-specific fashion, and such a pattern was similar to that in GTEx brain but not in other GTEx tissues. Importantly, DNMgene–CVgene subnetworks were enriched with pathways of chromatin remodeling, MHC protein complex binding, and neurotransmitter activities. In summary, our results unveiled that both DNMgenes and CVgenes contributed to a core set of biologically important pathways and networks, and their interactions may attribute to the risk for schizophrenia. Our results also suggested a stronger biological effect of DNMgenes than CVgenes in schizophrenia.

## Introduction

Schizophrenia is a chronic and socially disabling disorder whose pathophysiology remains unsolved^1^. During the past decade, a large body of genetic and genomic studies have demonstrated that genetic components susceptible to schizophrenia are highly heterogeneous and may involve a wide spectrum of risk factors, including common, rare, and de novo variants with effect sizes ranging from small to large^2–4^. Common variants (CVs), which are mainly investigated through genome-wide association studies (GWAS), and de novo mutations (DNMs), which are mainly discovered through next-generation sequencing of family trios, are two major groups of genetic variants. So far, more than a hundred CVs have been reported to be associated with schizophrenia^5–8^. CVs individually have small effect and, in combination, they explained a moderate proportion of the heritability of schizophrenia (less than 2%)^9^. Secondary analyses of GWAS data have revealed genomics and functional characteristics of CVs, highlighting their regulatory roles^3,10,11^ and enrichment in biological pathways^12–14^ and networks^15,16^. In contrast to CVs, DNMs are considered to have large effects and could presumably replenish the genetic variants that are wiped out by natural selection^17,18^. Under this hypothesis, complex disease like schizophrenia could keep a stable prevalence in population^19,20^, despite their reduced fecundity. Indeed, elevated rates of damaging DNMs have been reported in schizophrenia patients when compared to the unaffected controls^18,21^, as well as in other psychiatric disorders including autism^3^ and severe intellectual disability^22^. DNMs are highly heterogeneous, with a small number of DNMs recurrently found in more than one schizophrenia patient^23^. Except a few DNMs occurred in known schizophrenia candidate genes (e.g., *GRIN2A*^24^, *NRXN1*^25^, and *SHANK3*^26^), the majority have unknown implications for schizophrenia.

While insightful but inclusive results have reported for CVs and DNMs separately, little effort has been made to investigate whether and how these two types of variants interact at the gene, pathway, and network levels and share the contribution to disease onset or progression. We hypothesize that it is unlikely that these variants act independently, or through unrelated biological processes, to cause the disease. Accordingly, we hypothesize that these variants likely share functions in common biological pathways or processes. In this work, we examined the features of genes harboring DNMs (denoted as DNMgenes) and genes with CVs (denoted as CVgenes) based on evolutionary and network measurements. We introduced a schizophrenia gene network that could optimally link DNMgenes and CVgenes through co-expression-weighted protein–protein interactions (PPIs), where the co-expression profiles were obtained using spatiotemporal brain expression data or tissue-specific expression data.

## Methods

### DNM data

We downloaded the DNMs from *NPdenovo*^23^ for schizophrenia. *NPdenovo* is a recently developed database that curates DNMs from thousands of trios across multiple types of neuropsychiatric disorders including schizophrenia. The likelihood of each gene that contributes to the corresponding diseases was pre-calculated by the TADA program^27^ and the *p*-values were available on the *NPdenovo* website. After filtering by the *p*-value and expression profile (see Figure S1 for detailed filtering steps), we obtained a total of 254 genes with *p*-value < 0.05 in schizophrenia and were also expressed in at least one spatiotemporal site (see below, “Brain expression data”), referred as DNMgenes. Among them, one DNMgene (*LAMA2*) had three DNMs, eight genes had two DNMs (*TAF13*, *ESAM*, *RB1CC1*, *MKI67*, *PHF7*, *NIPAL3*, and *LPHN2*, ordered by TADA *p*-value), and the remaining genes had one DNM.

### CVs from GWAS data

The summary GWAS data were downloaded from the Psychiatric Genomics Consortium^5^. We selected variants with *p*-value < 5 × 10^−^^8^ and mapped them to genes if a variant was located in the gene body or within gene boundaries, which were defined as −35 kb upstream or downstream of each gene^12^. After requiring genes to be expressed in at least one spatiotemporal site, we identified 410 genes and referred them as CVgenes. In further analyses, we separated the CVgenes into 7 subgroups by their –log10(*p*) values (ranged between 7.30 and 29.75) and each required group has roughly similar number of genes (median 60, ranged between 34 and 83).

### Gene expression data

Schizophrenia is commonly considered as a brain disorder. Therefore, we downloaded a comprehensive brain expression dataset from BrainSpan Atlas^28^, which contained gene expression for multiple brain regions in multiple developmental stages. Following a previous work^29^, we grouped the samples into 12 categories based on their distinctive spatial and temporal features, ranging in three developmental stages (stage 1 (ST1), stage 2 (ST2), and stage 3 (ST3)) and four brain regions (FC: frontal cortex; SC: sub-cortical regions; SM: sensory-motor regions; and TP: temporal–parietal cortex) (Table S1). At each spatiotemporal site, we considered a gene was expressed if it had its RPKM (Reads Per Kilobase of transcript per Million mapped reads) value greater than one in one or more samples. We tested more stringent cutoff values, but the results in our following analyses were similar.

Tissue-specific gene expression data were downloaded from GTEx (version 6) to investigate the tissue-specific expression patterns^30^. A total of 27 tissues were considered, each with ≥30 samples. For each gene, we defined a *z*-score to measure its tissue specificity: $z_i\, = \,\frac{{expr_i\, - \,mean(Expr)}}{{sd(Expr)}}$, where $expr_i$ is the average gene expression of the gene in the *i*th tissue, *Expr* represents the collection of its average gene expression in all tissues, and $sd$ is the standard deviation of *Expr*. A higher *z*-score indicates the gene to be more specifically expressed in the investigated tissue.

### PPI and CoPPI networks

We built the reference human PPI network by combining data from the Human Protein Reference Database^31^ and the STRING^32^ database (hereafter referred as the HS network)^33^. After removing self-interactions and isolated nodes, the final HS network included 10,314 nodes (i.e., proteins) and 51,637 edges (i.e., interactions). A CoPPI is defined an edge-weighted PPI, in which each edge was weighted by the co-expression of the two nodes using the expression data generated for the specific spatiotemporal site. We used the absolute value of Pearson Correlation Coefficient (PCC) to measure the co-expression level between a pair of nodes. Edges involving unexpressed nodes were removed from the network.

### Network characteristics

We utilized three measurements in network analysis: node degree, shortest path, and betweenness centrality. Node degree is defined as the number of direct interactors of a node. The shortest path between two nodes was measured as the minimum length required for one node to traverse to another node in the network. The betweenness centrality measures the importance of a node. It is calculated by the number of shortest paths going through a node in the network. A high betweenness centrality value indicates that the corresponding node has a strong influence on the transfer of information in the network.

### Construction of subnetworks to link DNMgenes and CVgenes in spatiotemporal CoPPIs

To build a subnetwork that links the largest number of DNMgenes and CVgenes in the context of CoPPIs, we developed the following method. In a given CoPPI, we defined the union of DNMgenes and CVgenes in the HS network as the seed genes, i.e., genes of our interest. Our ultimate aim was to link seed genes using linker genes in a reference CoPPI. Theoretically, the linker genes can be any genes in the reference network except the seed genes. An exhaustive search within a large network would be very time consuming; therefore, we introduced two parameters ($r_1$ and $r_2$) to optimize our search of candidate linker genes with high probability. We first collected all other nodes that interacted with at least two genes of interest. For each of these candidate nodes (denoted by $canN$), we define $r_1\, = \,\frac{{mean(e_{canN\_intN})}}{{mean(e_{canN})}}$, where $intN$ denotes our genes of interest and *e* is the edge weight. Here $mean(e_{canN\_intN})$ is the average weight of edges between a candidate gene and a gene of our interest and $mean(e_{canN})$ is the average weight of all edges that the candidate gene is involved. Thus, $r_1$ measures the co-expression specificity between a candidate gene and our genes of interest. To control the impact of nodes with high degree in the network, we define $r_2\, = \,r_1\, \times \frac{{\# (interactors\mathop { \cap }\nolimits intN)}}{{\# interactor}}$ for each candidate node, where $\frac{{\# (interactors\mathop { \cap }\nolimits intN)}}{{\# interactor}}$ measures the specificity of a candidate gene’s interactors overlapping with our genes of interest. Based on our parameter evaluation, we applied $r_1 \ge 1.2$ and $r_2 > 0.1$ to our candidate gene selection (see Results). These thresholds of $r_1$ and $r_2$ resulted in selection of ~25% most promising nodes in the network being candidate genes and ~5–10% nodes interacting with two or more DNMgenes or CVgenes. We give a subnetwork score $s = r_3 \times mean(e_{subnetwork})$, where $r_3 = \frac{{\# linked(DNMgenes,CVgenes)}}{{\# union(DNMgenes,CVgenes)}}$. Here, $r_3$ measures the proportion of DNMgenes and CVgenes that are connected in the subnetwork. Our aim is to link the DNMgenes and CVgenes in the context of spatiotemporal (e.g., the 12 sets in brain expression) or tissue-specific (e.g., the GTEx data) CoPPI networks with a goal to maximize the proportion of linked seed genes (DNMgenes and CVgenes) and the within-subnetwork co-expression values. Starting with the seed genes, we iteratively choose nodes from the candidate gene list that can maximize *s*, until the increase ratio of *s* is less than 0.5%. We applied the method to each of the 12 spatiotemporal points and 27 GTEx tissues. In each application, we also conducted a randomization test by selecting a random set of seed genes with the same size as our genes of interest. Starting with each random set of seed genes, we applied the method in the same way and using the same threshold set to maximize the connection of seed genes, resulting in 100 random subnetworks. The code to construct the subnetworks is available at https://github.com/bsml320/VariantSubnetwork.

## Results

### Temporal and spatial expression patterns of DNMgenes and CVgenes

**Fig. 1:**
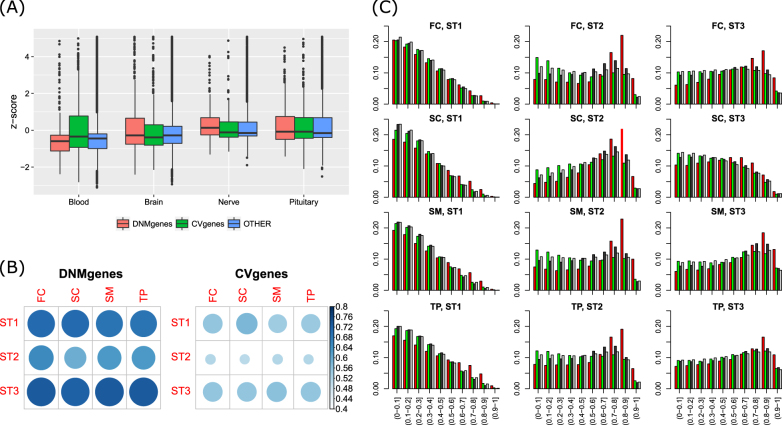
Temporal and spatial expression patterns of DNMgenes and CVgenes. **a** Tissue-specific gene expression of the three gene sets using the three tissues that are related to psychiatric diseases (brain, nerve, and pituitary) and the blood tissue as control. **b** Proportion of DNMgenes (left) or CVgenes (right) that were expressed in 12 spatiotemporal points ranging in three developmental stages (stage 1 or ST1, stage 2 or ST2, and stage 3 or ST3) and four brain regions (FC frontal cortex, SC sub-cortical regions, SM sensory-motor regions, and TP temporal-parietal cortex). **c** Co-expression patterns among DNMgenes (red), CVgenes (green), random genes with the sample size of the union set of DNMgenes and CVgenes (black), and all genes (gray) in 12 spatiotemporal points. *X*-axis: average co-expression of the investigated genes, *y*-axis: proportion of genes for the particular gene set

To test the tissue-specific expression of these genes, we selected three tissues that are related to psychiatric diseases (brain, nerve, and pituitary) and selected the blood tissue as a control for analyses. All four tissues were from GTEx data. As shown in Fig. 1a, DNMgenes showed significantly higher tissue-specific expression in nerve (*p* = 6.39 × 10^−4^, *p*_BH_ = 7.67 × 10^−3^, one-sided *t*-test comparing DNMgenes versus non-DNMgenes in each tissue; multiple testing correction using the Benjamini & Hochberg (BH) method^34^ on all 27 tissues and 4 gene groups) but not in brain, pituitary, or blood. CVgenes showed no tissue-specific expression in any of these tissues; the same for each subgroup of CVgenes (Figure S2). In addition, by analyzing the spatiotemporal brain expression data, we observed that both DNMgenes and CVgenes tended to be expressed in stages 1 and 3 than in stage 2. Particularly, 69.0–71.0% DNMgenes were expressed in stage 1 and 72.6–73.4% DNMgenes were expressed in stage 3, compared to 59.5–65.0% DNMgenes expressed in stage 2 (Fig. 1b). Meanwhile, 53.9–58.3% CVgenes were expressed in stage 1 or stage 3, much higher than in stage 2 (50.5–52.0%). A heatmap of both groups of genes in 12 spatiotemporal points is presented in Figure S3. Comparing co-expression patterns of each group of genes, we found a significant overload of high co-expression among DNMgenes in stages 2 and 3 (Fig. 1c). Such a pattern of DNMgenes was universally observed in all four brain regions. In contrast, CVgenes did not show significant co-expression in any stage or any region (Fig. 1c).

### Network characteristics of DNMgenes and CVgenes

**Fig. 2:**
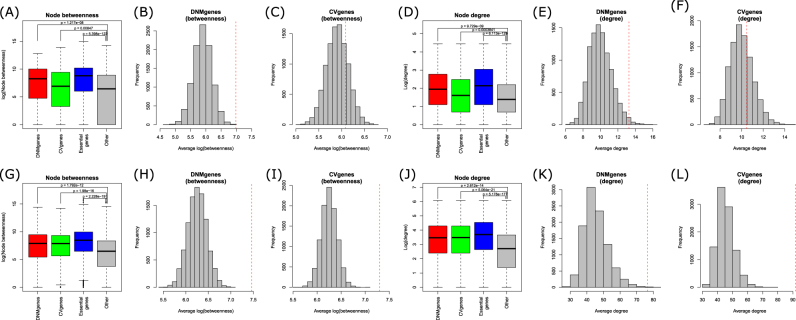
Network characteristics of four gene sets: DNMgenes, CVgenes, essential genes, and other genes. **a** Distribution of the node betweenness in the HS protein–protein interaction network. **b**, **c** Randomization test of the betweenness for DNMgenes (**b**) and CVgenes (**c**) in the HS network. The dotted red line indicates the actual betweenness of DNMgenes (**b**) or CVgenes (**c**) in the HS network. **d** Comparison of the node degree. **e**, **f** Randomization test of the node degree for DNMgenes (**e**) and CVgenes (**f**). **g**–**i** Distribution and randomization test of the node betweenness in the PathwayCommons (PC) network. **j**–**l** Distribution and randomization test of the node degree in the PC network

Characteristics of genes in PPI networks may reflect their functional importance. We first examined the betweenness centrality of the four gene sets in the HS network. DNMgenes had significantly higher betweenness values (*n* = 177 DNMgenes, average log(betweenness): 6.982, *p* = 1.217 × 10^−8^, Wilcoxon Rank-Sum test) than other genes (*n* = 7932, average: 5.450) in the HS network, but CVgenes (*n* = 274, average: 6.083, *p* = 8.47 × 10^−3^) showed only marginal significance (Fig. 2a–c). We also conducted a randomization test by selecting the same number of DNMgenes or CVgenes from the network 10,000 times and calculated an empirical *p*-value as the proportion of random gene sets exceeding the average betweenness in DNMgenes or CVgenes, respectively. This randomization test proved that the observed betweenness of DNMgenes was significantly higher than randomly expected (*p*_empirical_ < 1 × 10^−4^), but the observed betweenness of CVgenes failed in the randomization test (*p*_empirical_ = 0.222).

Node degree, also called node connectivity, measures the number of direct interactors of a node in a network. A node with a high degree often implies important functions in a biological system. DNMgenes had substantially high node degree (average = 13.26), nearly twice of the other genes (average: 7.90; *p* = 9.73 × 10^−9^, Wilcoxon Rank-Sum test, Fig. 2d; *p*_empirical_ = 0.014, the randomization test, Fig. 2e). For CVgenes, although we observed statistically higher node degree (average: 10.47, *p* = 3.84 × 10^−4^) than other genes, its *p*-value became insignificant after the randomization test (*p*_empirical_ = 0.310, Fig. 2f). As a comparison, essential genes had the highest degree (average: 18.32), which was significantly higher than the other genes (*p* = 6.11 × 10^−129^).

To further validate our results, we conducted the same analyses in the reference network from PathwayCommons (PC)^35^, which encompasses various sources of PPIs including both physical interactions and interactions in signaling pathways. In the PC network, we confirmed that both DNMgenes and CVgenes had significantly higher betweenness and higher node degree values than other genes (Fig. 2g–l), all of which were validated in randomization tests. A trend toward higher betweenness values was also observed in most subgroups of CVgenes (Figure S4), while the high node degree values of CVgenes were mainly driven by those with strongest statistical significance, i.e., the subgroups whose –log10(*p*) values were between 18 and 30 (Figure S4).

### Connections between DNMgenes and CVgenes were conditionally insignificant

**Fig. 3:**
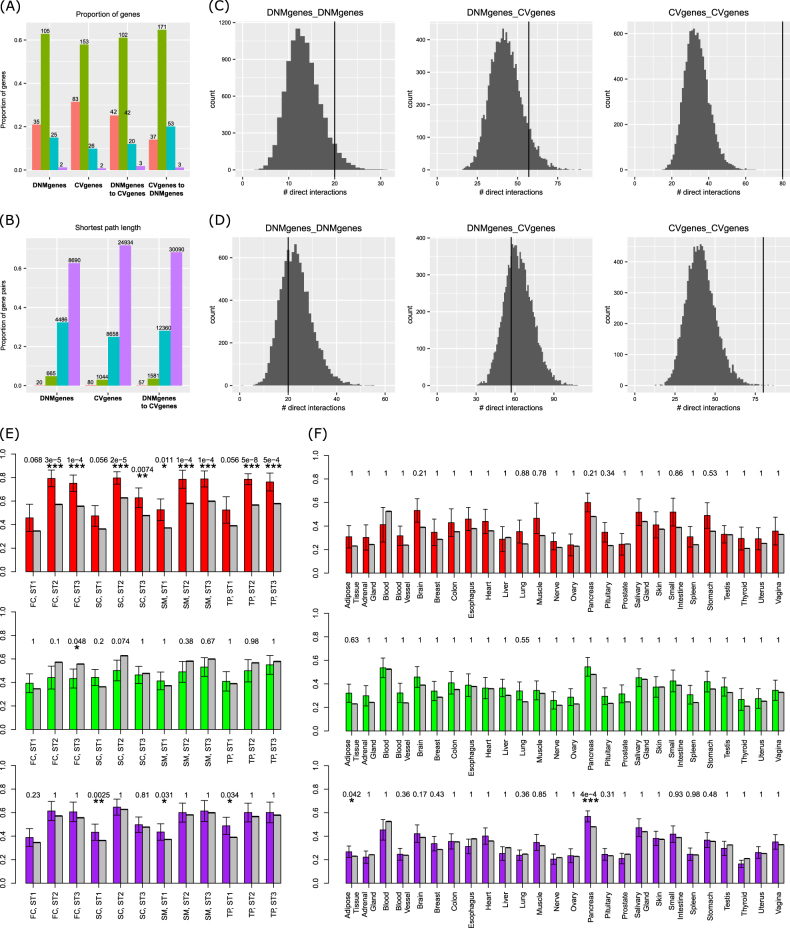
Connections between DNMgenes and CVgenes. **a**, **b** Distribution of the proportion of genes that can be directly connected (**a**) and the proportion of gene pairs stratified by the shortest path among them (**b**). **c**, **d** Randomization test for the direct interactions among DNMgenes, between DNMgenes and CVgenes, and among CVgenes. In **c**, the randomization test was conducted using 10,000 random sets with the same number of genes in the corresponding test settings. In **d**, the randomization test was conducted using 10,000 random sets that have matched degree distribution as in the actual case. **e** Co-expression of the edges between DNMgenes (red bars in the top panel), between CVgenes (green bars in the middle panel), and between DNMgenes and CVgenes (purple bars in the bottom panel). In all panels, gray bars indicate other edges excluding those in investigation; *t*-test was conducted to compare the co-expression levels between edges involved in a particular gene group and other edges. Adjusted *p*-values after Bonferroni correction were labeled for each test (***p_adjust_ < 0.001; **p_adjust_ < 0.01; *p_adjust_ < 0.05). *Y*-axis: average co-expression as measured by absolute Pearson Correlation Coefficient. **f** Co-expression of the direct interactions among DNMgenes, among CVgenes, and between DNMgenes and CVgenes in GTEx tissues

Several studies have previously proved that DNMgenes tended to interact with each other more often than randomly expected^29^. We first explored the interactions and shortest paths between two individual DNMgenes, two individual CVgenes, and any of a DNMgene and a CVgene. For the 254 DNMgenes (177 in the HS network) and 410 CVgenes (274 in the HS network), there were only 15 genes overlapped and ten were in the HS network: *ALAS1*, *CACNA1I*, *CUL3*, *FKBPL*, *GRIN2A*, *HIST1H1E*, *LRP1*, *SGSM2*, *STAG1*, and *SYNGAP1*. To explore the shortest path among different groups of genes, we excluded these ten genes from each group. As a result, we observed that 35 DNMgenes directly interacted with each other through 20 edges (Fig. 3a, b), which were nominally significantly higher than random gene sets in the HS network (*p* = 0.047, Fig. 3c, top panels) and significant in the PC network (*p* < 1 × 10^−4^, data not shown in Fig. 3). CVgenes significantly interact with each other more often than the random expectation in both the HS network (*p* < 1 × 10^−4^) and the PC network (*p* < 1 × 10^−4^). The PPIs between DNMgenes and CVgenes were marginally significant in the HS network (*p* = 0.079) but significant in the PC network (*p* < 1 × 10^−4^). Collectively, these results implied that DNMgenes and CVgenes significantly interacted within their groups and with each other.

However, considering that both DNMgenes and CVgenes had higher degree values than the other genes, we conducted a conditional resampling by requiring the random sets to have the same degree distribution. To this end, we ordered the nodes in the whole HS network according to their node degree and categorized them into four groups with approximately equal sizes. For each random set, we choose the same number of DNMgenes from each of the four degree groups when evaluating the edges between DNMgenes, and similarly applied to the edges between CVgenes or the edges between DNMgenes and CVgenes. In this conditional resample analysis, the overrepresentation of PPIs within the CVgene group remained significant in both the HS network (*p* = 5 × 10^−4^) and the PC network (*p* < 1 × 10^−4^). However, the PPIs within the DNMgene group (*p* = 0.647) or between DNMgenes and CVgenes (*p* = 0.673) were no longer significantly higher than random expectation (Fig. 3c, bottom panels). This degree-matched randomization test thus implied that we should be cautious when interpreting the interactions among DNMgenes or between DNMgenes and CVgenes.

The interactions between two individual DNMgenes, between two individual CVgenes, and between any of a DNMgene and a CVgene all displayed different co-expression trend in a spatiotemporal way. Particularly, PPI pairs between DNMgenes tended to be significantly highly co-expressed than other PPI pairs in the HS network in all 12 spatiotemporal points (Fig. 3d). Interestingly, CVgenes showed opposite trend of co-expressed PPIs in stage 1 and stages 2–3 and were only marginally significant. PPIs between DNMgenes and CVgenes were significantly highly co-expressed than other PPIs. However, none of the PPI groups had tissue-specific co-expression as evaluated using the GTEx data (Fig. 3e).

### Building subnetworks enriched with DNMgenes and CVgenes

**Fig. 4:**
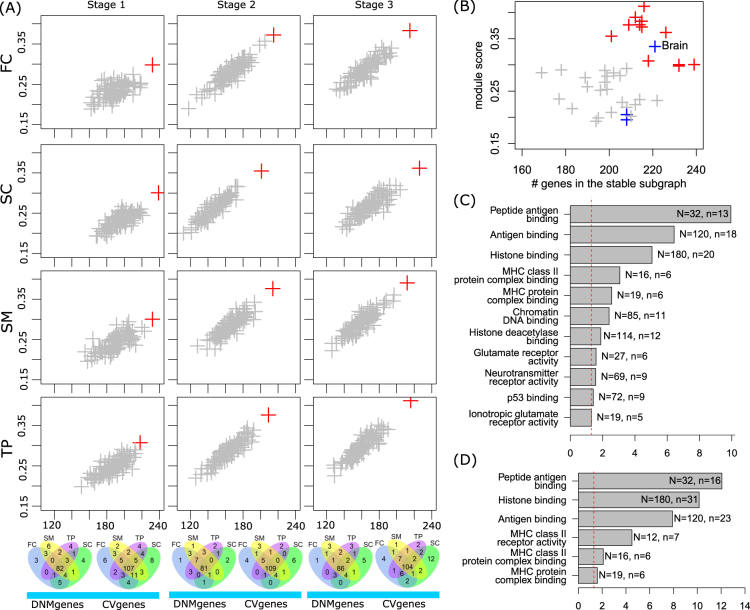
Schizophrenia subnetwork analysis. **a** Comparison of the stable subnetwork obtained in each of the 12 spatiotemporal points (red) with 100 random subnetworks (gray). *X*-axis: the number of genes of interest (i.e., the union of DNMgenes and CVgenes) in the stable subnetwork; *y*-axis: score of the subnetwork (see main text for details). In each panel, the 100 random subnetworks were identified using random seed genes in the same size as our genes of interest and were obtained using the same method. Comparison of the DNMgenes and CVgenes in the stable subnetwork is shown in Venn diagrams. **b** Comparison of the 12 subnetworks obtained using 12 spatiotemporal brain data from BrainSpan Atlas (red) with subnetworks obtained using GTEx tissue-specific expression. The GTEx brain, nerve, and pituitary are shown in blue while other tissues are shown in gray. **c** Gene Ontology enrichment analysis of component genes in FC, stage 1. Significantly enriched (*p*_Bonferoni_ < 0.05) Gene Ontology Molecular Function were obtained using ToppFun. **d** Gene Ontology enrichment analysis of the original genes of interest, i.e., the union of DNMgenes and CVgenes

To investigate the links between DNMgenes and CVgenes in cellular system, we developed a spatiotemporal network-assisted approach with an aim of identifying high co-expression and high connection between these two groups of genes. We applied the approach in each of the 12 spatiotemporal points (Fig. 4a) and in 27 GTEx tissues (Fig. 4b). We referred the subnetwork achieved in each case as the stable subnetwork. As shown in Fig. 4a, a larger number of genes were connected in stage 1 (232 in FC, 239 in SC, 232 in SM, and 218 in TP) than in other stages, whereas in stages 2 and 3, higher co-expression was achieved (*s* > 0.35). Importantly, the connected DNMgenes and CVgenes were largely overlapped in all regions and in each stage (bottom Venn diagram in Fig. 4a). In all 12 spatiotemporal points, the stable subnetworks (red cross in each panel in Fig. 4a) obtained using the actual data showed elevated co-expression and connection than those observed in 100 subnetworks (gray crosses in each panel in Fig. 4a) obtained using random seed genes. Notably, the 100 subnetworks in each case were not simply random subnetworks by matching the size. Only the seed genes were randomly selected from the network while the subnetworks were obtained using the same method. Thus, these 100 subnetworks were also optimized toward high co-expression and high connection between their random seed genes. When applying the same method in the 27 GTEx tissues, we observed a subnetwork with the most connected genes and the highest module score in brain. The connected seed genes and the co-expression level of this subnetwork from GTEx brain were comparable with the subnetworks from 12 spatiotemporal data (Fig. 4b).

In our subnetwork construction, we employed three parameters: *r*_1_ and *r*_2_ to determine the candidate genes being included in the subnetwork, and an increase ratio to control the stop of subnetwork expansion. We tested a range of these parameters, including *r*_1_ = [1.0, 1.1, 1.2, 1.3, 1.4, 1.5] and *r*_2_ = [0.05, 0.1, 0.15] (Figure S5). We finally chose $r_1 \ge 1.2$ and $r_2 > 0.1$ to have ~25% of nodes in the subnetwork being considered as candidate genes and ~5–10% nodes being interacted with two or more DNMgenes or CVgenes. These proportions were validated in all 12 spatiotemporal CoPPIs as well as 12 random seed gene sets (Figure S6). A different threshold for the increase ratio, 0.2%, was also tested. As shown in Figure S7, the same results were observed, that is, a larger number of genes were connected in the stable subnetwork than the size-matched random genes.

Functional enrichment analysis^36^ of the component genes in the stable subnetwork (FC, stage 1) highlighted histone binding genes (*p*_Bonferroni_ = 8.72 × 10^−6^), MHC class II protein complex binding (*p*_Bonferroni_ = 8.57 × 10^−4^), chromatin DNA binding (*p*_Bonferroni_ = 4.00 × 10^−3^), glutamate receptor activity (*p*_Bonferroni_ = 0.025), and neurotransmitter receptor genes (*p*_Bonferroni_ = 0.027) (Fig. 4c). Note that the histone and MHC genes were readily detectable in the original DNMgene and CVgene lists (Fig. 4d), while the neurotransmitter genes were only significant in our stable subnetwork. Genes with function in the neuronal transmitter activities were found including *AXIN1* (a link gene), *CNKSR2* (a CVgene), *DLG2* (a DNMgene), *DRD2* (a CVgene), *CACNA1C* (a CVgene), *EPB41L1* (a link gene), *GRID2* (a DNMgene), *GRIA1* (a CVgene), *GRIN2A* (a CVgene and a DNMgene), *GRIN1* (a link gene), *NRGN* (a CVgene), *RYR2* (a link gene), and *SYNGAP1* (a CVgene and a DNMgene). The gene sets, namely histone binding (mainly histone genes), chromatin DNA binding (*CHD4*, *EP300*, *FOXO3*, *HIST1H1B*, *HIST1H1C*, *HIST1H1E*, *MECP2*, *MTA2*, *SMARCC1*, *SMARCC2*, *SRF*), and histone deacetylase binding (*DDX20, DNMT1, GLI3, HIF1A, HIST1H1B, HSPA1A, HSPA1B, MAPK8, MECP2, MTA2, NIPBL, SRF*), can all be considered as chromatin remodeling pathways. The subnetwork in FC, stage 1 was shown in Figure S8 as a demonstration, where DNMgenes and CVgenes were all mixed together, implying that the two groups of genes are likely functionally connected.

## Discussion

The genetic architecture underlying schizophrenia has been proved as highly heterogeneous, involving a wide range of genetic variants. While each type of the variants is characterized with unique features in terms of effect sizes, causal roles, and functional interpretations, it is unlikely that different types of variants disrupt unrelated biological processes to cause the same diseases. In this work, we characterized genes harboring different types of variants and made links among these genes. We unveiled that both DNMgenes and CVgenes had comparable, evolutionary conservation levels and protein ages as essential genes, suggesting their critical functional importance. Although DNMgenes and CVgenes appeared sparsely connected, we found that they could be linked in a tissue-specific fashion. The subnetworks they formed had significantly higher co-expression levels than expected from random genes and displayed similar tissue co-expression pattern to brain but not the other tissues. Importantly, the network analysis showed the convergence of DNMgenes and CVgenes toward pathways of chromatin remodeling, MHC protein complex genes, and neuronal transmitter activities.

Although it has been widely accepted that both CVs and DNMs contribute to the genetic components of schizophrenia, it has been essentially unknown to us how they affect the function and lead to the diseases. Our results provided insights into the roles of CVgenes and DNMgenes in schizophrenia. The DNMs considered in our work included truncation mutations and deleterious missense mutations, both of which were expected with severe impact on protein products. In contrast, most CVs are located in non-coding regions while increasing lines of evidence have indicated that these variants play regulatory roles on target gene expression levels to contribute to diseases^37,38^. The fact that DNMgenes have higher betweenness indicates that DNMgenes themselves have important roles in the networks, because many paths in the network go through these DNMgenes according to the definition of betweenness. Removal of these genes, e.g., through nonsense mutations, would likely result in severe impact on the whole network.

While the previous findings of CVs (CVgenes) and DNMs (DNMgenes) are important for future studies, some of these mutations (genes) do not necessarily contribute to schizophrenia. Therefore, selection of these candidate mutations/genes is critical in our future investigation of their risk to schizophrenia. In our study, we hypothesize that these two types of variants (and genes) will share similar biological processes and interact in the network. With this rationale, our approach may pinpoint more promising candidate genes for schizophrenia, as well as their possible molecular mechanisms. Our results resembled risk factors critical to pathogenesis of schizophrenia that had been previously implicated. Genes of the MHC complex^12,39^ and regulation of neurogenesis have long been implicated in schizophrenia, while genes from chromatin remodeling pathways have been recently reported^40^. Growing evidence has suggested that chromatin organization, especially epigenetic dysregulation, is likely an important mechanism in the pathogenesis of schizophrenia. Genes reported in GWAS results, which function in epigenetic regulation, are mainly histone genes. Genes with DNMs in schizophrenia patients, such as *CHD2*, *MECP2*, and *HUME1*, have converged molecular functions in epigenetic regulation of transcription^18^. Recently, a large-scale whole-exome sequencing study using >4000 schizophrenia and >1000 trios revealed the gene *SETD1A* as a risk gene for schizophrenia^40^, which further proved the potential roles of chromatin organization.

To provide additional biological insights, we also investigated the evolutionary features of DNMgenes and CVgenes (Figure S9). We used the dN/dS ratio and the evolutionary rate, both of which are commonly used for studying molecular evolution and inferring the functional importance. Our results showed that DNMgenes and CVgenes had significantly low dN/dS ratio and low evolutionary rate compared to other genes. In addition, we found that DNMgenes and CVgenes were significantly older than other genes, but similar to essential genes, as measured by the average protein age. Notably, DNMgenes had the oldest age among the four gene groups (DNMgenes, CVgenes, essential genes, and other genes). Because previous reports have shown that disease genes tended to be ancient^41–43^, these results indicated that DNMgenes and CVgenes likely had critical functions.

In conclusion, we studied CVs and DNMs in schizophrenia using evolutionary measurements, the human PPI network, and disease-relevant spatiotemporal co-expression networks. Our results revealed different patterns of genes harboring the two types of variants. These genes, although appeared distant, were more accessible to each other and formed a convergent network enriched in three functional groups. Future validation will warrant the impact of our work.

## Electronic supplementary material

Supp File 1

## References

[1] Sullivan PF, Daly MJ, O’Donovan M (2012). Genetic architectures of psychiatric disorders: the emerging picture and its implications. Nat. Rev. Genet..

[2] Allen NC (2008). Systematic meta-analyses and field synopsis of genetic association studies in schizophrenia: the SzGene database. Nat. Genet..

[3] Richards AL (2012). Schizophrenia susceptibility alleles are enriched for alleles that affect gene expression in adult human brain. Mol. Psychiatry.

[4] Rees E, Kirov G, O’Donovan MC, Owen MJ (2012). De novo mutation in schizophrenia. Schizophr. Bull..

[5] Schizophrenia Working Group of the Psychiatric Genomics Consortium. (2014). Biological insights from 108 schizophrenia-associated genetic loci. Nature.

[6] Shi J (2009). Common variants on chromosome 6p22.1 are associated with schizophrenia. Nature.

[7] International Schizophrenia C (2009). Common polygenic variation contributes to risk of schizophrenia and bipolar disorder. Nature.

[8] Stefansson H (2009). Common variants conferring risk of schizophrenia. Nature.

[9] Cross-Disorder Group of the Psychiatric Genomics Consortium. (2013). Identification of risk loci with shared effects on five major psychiatric disorders: a genome-wide analysis. Lancet.

[10] Bacanu SA (2014). Functional SNPs are enriched for schizophrenia association signals. Mol. Psychiatry.

[11] Roussos P (2014). A role for noncoding variation in schizophrenia. Cell Rep..

[12] The Network and Pathway Analysis Subgroup of the Psychiatric Genomics Consortium. (2015). Psychiatric genome-wide association study analyses implicate neuronal, immune and histone pathways. Nat. Neurosci..

[13] O’Dushlaine C (2011). Molecular pathways involved in neuronal cell adhesion and membrane scaffolding contribute to schizophrenia and bipolar disorder susceptibility. Mol. Psychiatry.

[14] Jia P, Wang L, Meltzer HY, Zhao Z (2010). Common variants conferring risk of schizophrenia: a pathway analysis of GWAS data. Schizophr. Res..

[15] Jia P (2012). Network-assisted investigation of combined causal signals from genome-wide association studies in schizophrenia. PLoS Comput. Biol..

[16] Wang Y (2016). Leveraging genomic annotations and pleiotropic enrichment for improved replication rates in schizophrenia GWAS. PLoS Genet..

[17] Xu B (2011). Exome sequencing supports a de novo mutational paradigm for schizophrenia. Nat. Genet..

[18] McCarthy SE (2014). De novo mutations in schizophrenia implicate chromatin remodeling and support a genetic overlap with autism and intellectual disability. Mol. Psychiatry.

[19] Purcell SM (2014). A polygenic burden of rare disruptive mutations in schizophrenia. Nature.

[20] Fromer M (2014). De novo mutations in schizophrenia implicate synaptic networks. Nature.

[21] Girard SL (2011). Increased exonic de novo mutation rate in individuals with schizophrenia. Nat. Genet..

[22] de Ligt J (2012). Diagnostic exome sequencing in persons with severe intellectual disability. New Engl. J. Med..

[23] Li J (2016). Genes with de novo mutations are shared by four neuropsychiatric disorders discovered from NPdenovo database. Mol. Psychiatry.

[24] Tarabeux J (2011). Rare mutations in N-methyl-D-aspartate glutamate receptors in autism spectrum disorders and schizophrenia. Transl. Psychiatry.

[25] Todarello G (2014). Incomplete penetrance of NRXN1 deletions in families with schizophrenia. Schizophr. Res..

[26] Gauthier J (2010). De novo mutations in the gene encoding the synaptic scaffolding protein SHANK3 in patients ascertained for schizophrenia. Proc. Natl Acad. Sci. USA.

[27] He X (2013). Integrated model of de novo and inherited genetic variants yields greater power to identify risk genes. PLoS Genet..

[28] BrainSpan Atlas. http://www.brainspan.org/.

[29] Gulsuner S (2013). Spatial and temporal mapping of de novo mutations in schizophrenia to a fetal prefrontal cortical network. Cell.

[30] Mele M (2015). Human genomics. The human transcriptome across tissues and individuals. Science.

[31] Keshava Prasad TS (2009). Human Protein Reference Database–2009 update. Nucleic Acids Res..

[32] Szklarczyk D (2011). The STRING database in 2011: functional interaction networks of proteins, globally integrated and scored. Nucleic Acids Res..

[33] Hormozdiari F, Penn O, Borenstein E, Eichler EE (2015). The discovery of integrated gene networks for autism and related disorders. Genome Res..

[34] Benjamini Y, Hochberg Y (1995). Controlling the false discovery rate: a practical and powerful approach to multiple testing. J. Royal Stat. Soc. Ser. B.

[35] Cerami EG (2011). Pathway Commons, a web resource for biological pathway data. Nucleic Acids Res.

[36] Chen J, Bardes EE, Aronow BJ, Jegga AG (2009). ToppGene Suite for gene list enrichment analysis and candidate gene prioritization. Nucleic Acids Res..

[37] Jiang J, Jia P, Shen B, Zhao Z (2014). Top associated SNPs in prostate cancer are significantly enriched in cis-expression quantitative trait loci and at transcription factor binding sites. Oncotarget.

[38] Nicolae DL (2010). Trait-associated SNPs are more likely to be eQTLs: annotation to enhance discovery from GWAS. PLoS Genet..

[39] Xu J (2012). RNA-Seq analysis implicates dysregulation of the immune system in schizophrenia. BMC Genomics.

[40] Singh T (2016). Rare loss-of-function variants in SETD1A are associated with schizophrenia and developmental disorders. Nat. Neurosci..

[41] Cheng F (2014). Studying tumorigenesis through network evolution and somatic mutational perturbations in the cancer interactome. Mol. Biol. Evol..

[42] Domazet-Loso T, Tautz D (2008). An ancient evolutionary origin of genes associated with human genetic diseases. Mol. Biol. Evol..

[43] Maxwell EK (2014). Evolutionary profiling reveals the heterogeneous origins of classes of human disease genes: implications for modeling disease genetics in animals. BMC Evol. Biol..

