# Supplementary material for: Convergent roles of de novo mutations and common variants in schizophrenia in tissue-specific and spatiotemporal co-expression network
Source: Transl Psychiatry. 2018 May 24;8:105. doi: 10.1038/s41398-018-0154-2 (PMC5967316; doi:10.1038/s41398-018-0154-2)
Supplement: Supplementary file 1 — Supp File 1 [file 41398_2018_154_MOESM1_ESM.docx]

**
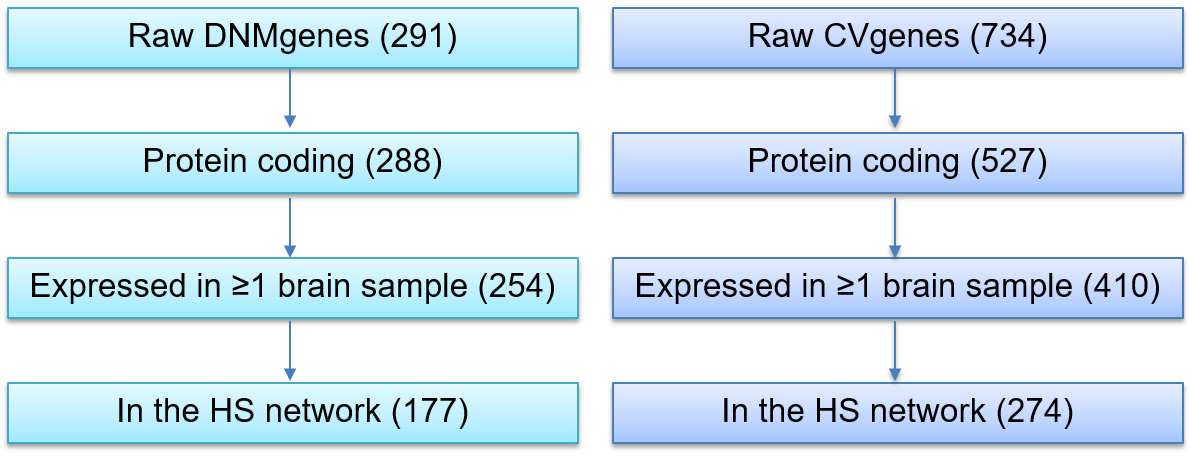
**

**Figure S1**. Data preparation pipeline to collect DNMgenes and CVgenes.


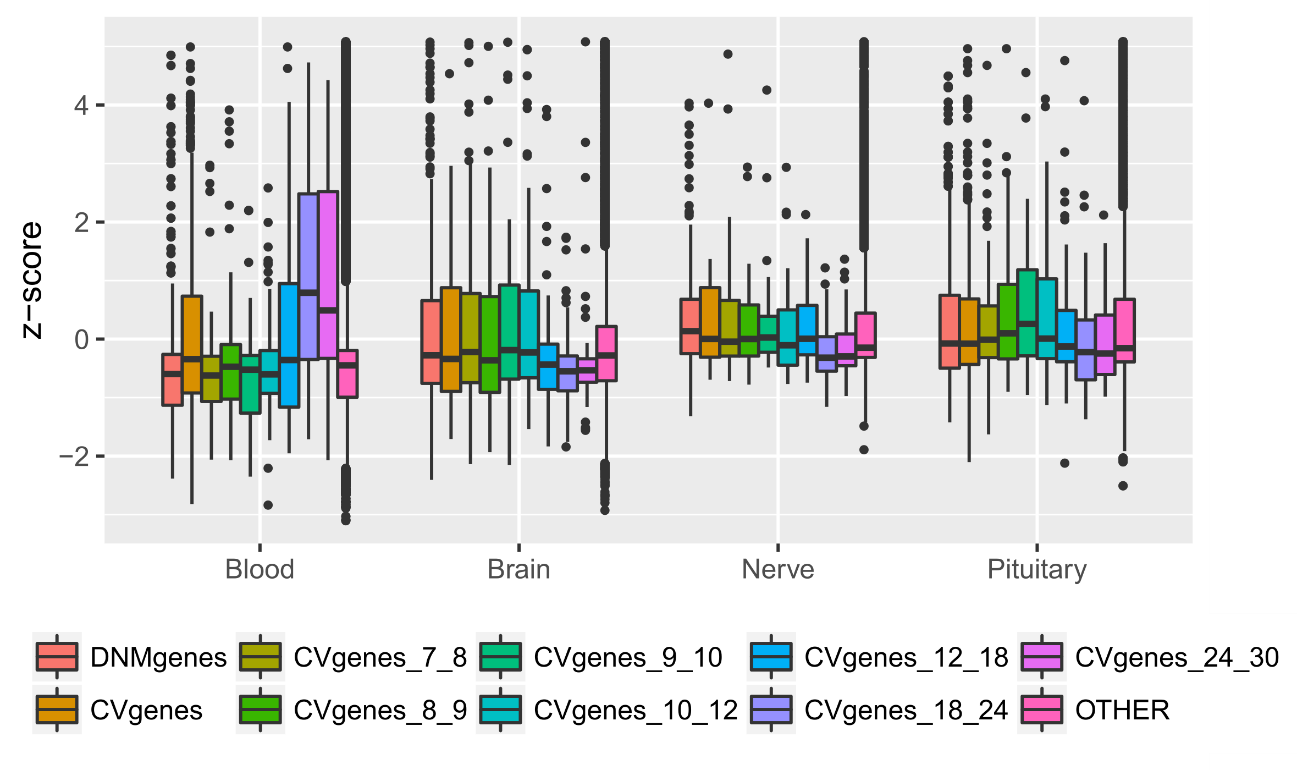


**Figure S2.** Tissue-specific gene expression of different gene sets using the three tissues that are related to psychiatric diseases (brain, nerve, and pituitary) and the blood tissue as control. CVgenes_7_8: CVgenes whose –log10(p) fall between 7 and 8, corresponding to 5×10^-8^< p ≤ 1×10^-8^. CVgenes_8_9: CVgenes whose –log10(p) fall between 8 and 9, corresponding to 1×10^-8^< p ≤ 1×10^-9^. CVgenes_9_10: CVgenes whose –log10(p) fall between 9 and 10, corresponding to 1×10^-9^< p ≤ 1×10^-10^. CVgenes_10_12: CVgenes whose –log10(p) fall between 10 and 12, corresponding to 1×10^-10^< p ≤ 1×10^-12^. CVgenes_12_18: CVgenes whose –log10(p) fall between 12 and 18, corresponding to 1×10^-12^< p ≤ 1×10^-18^. CVgenes_18_24: CVgenes whose –log10(p) fall between 18 and 24, corresponding to 1×10^-18^< p ≤ 1×10^-24^. CVgenes_24_30: CVgenes whose –log10(p) fall between 24 and 30, corresponding to 1×10^-24^< p ≤ 1×10^-30^.

**
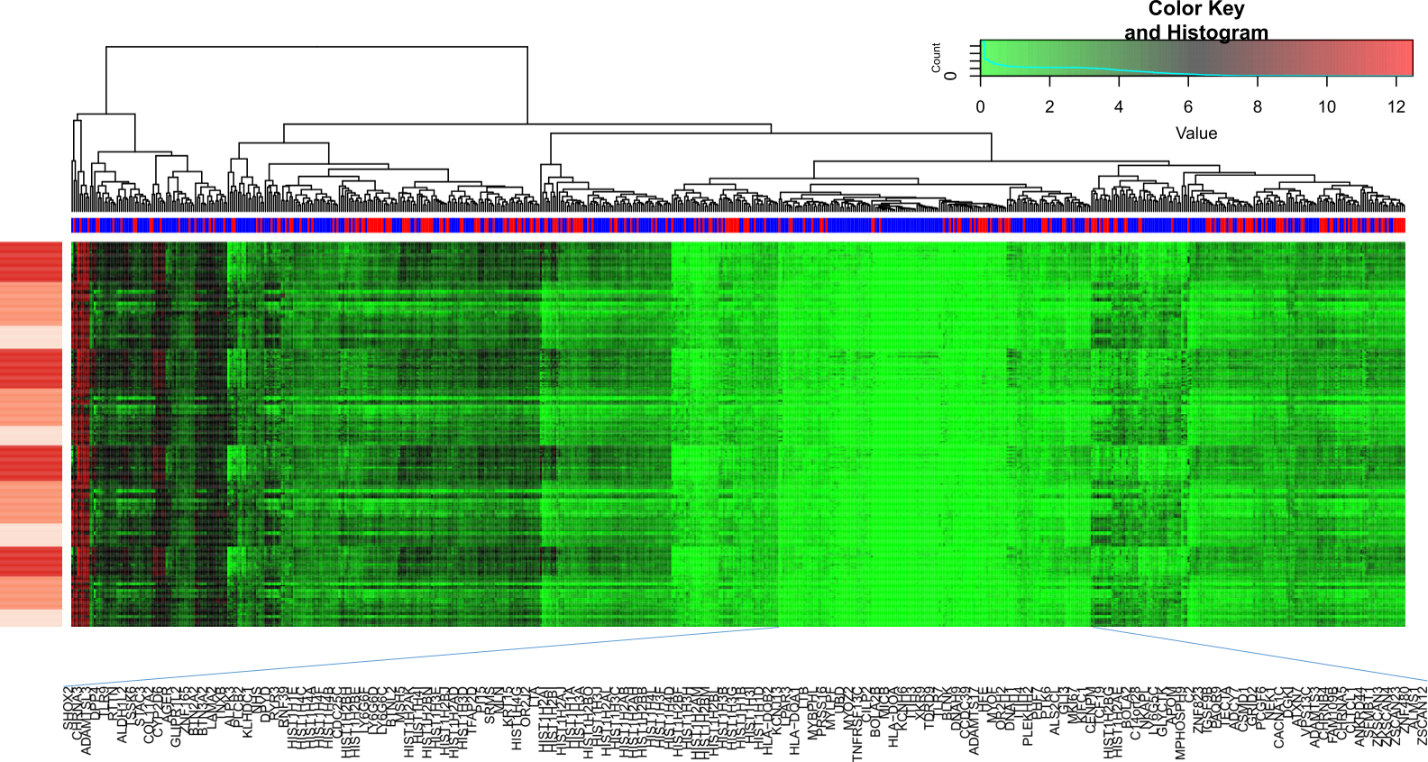
**

**Figure S3**. Heatmap of the original DNMgenes and CVgenes in all 12 spatiotemporal points.

**
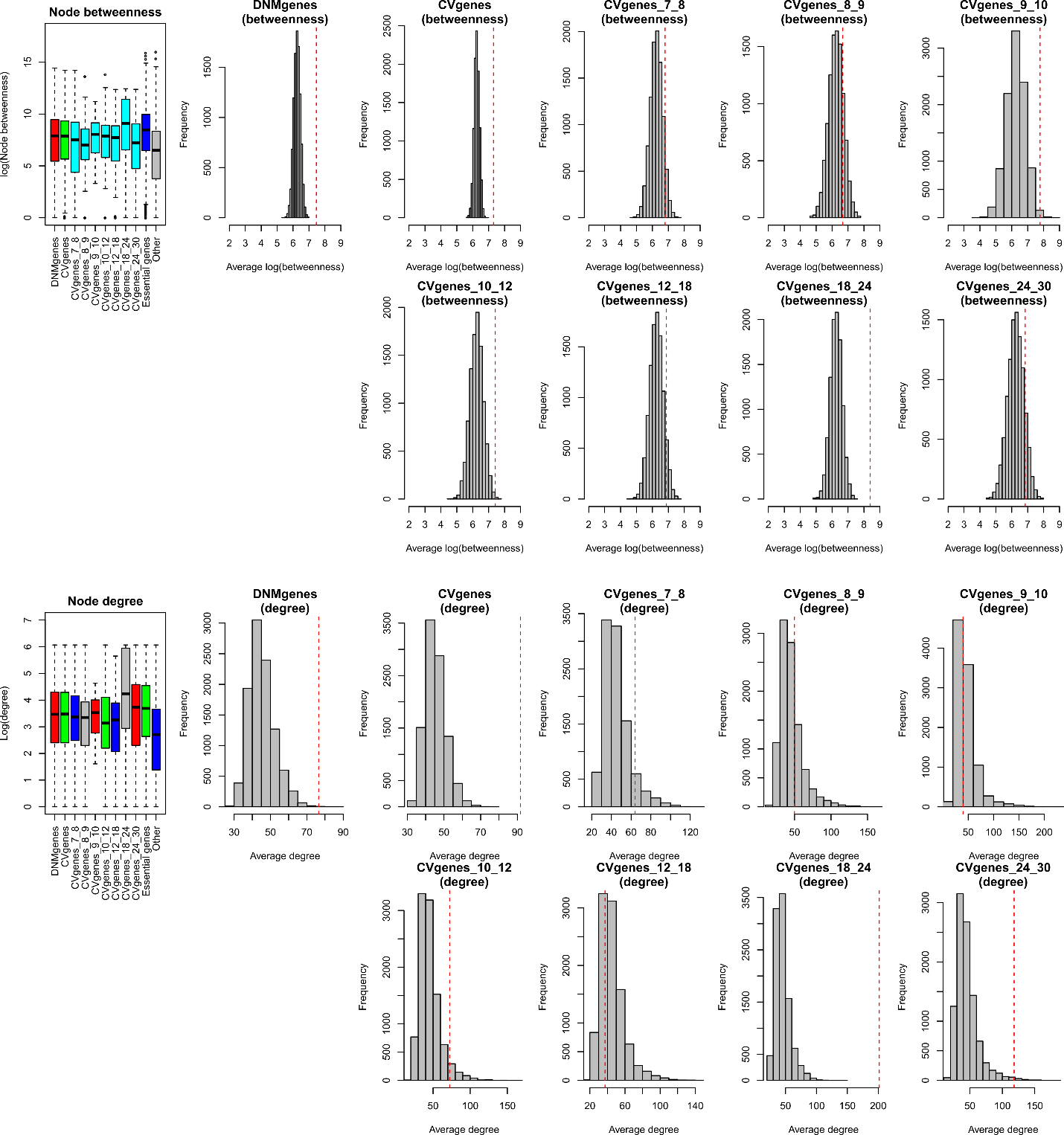
**

**Figure S4.** Network characteristics of different gene sets using the PathwayCommons (PC) network. The boxplots showed the distribution of betweenness (top) or node degree. The histograms illustrated the randomization test. In each histogram, 10,000 random sets of genes with the matched set size of the corresponding group were selected and estimated for their betweenness or node degree. The red dot lines indicated the actual value for the gene group. Definition of gene groups can be found in Figure S2.


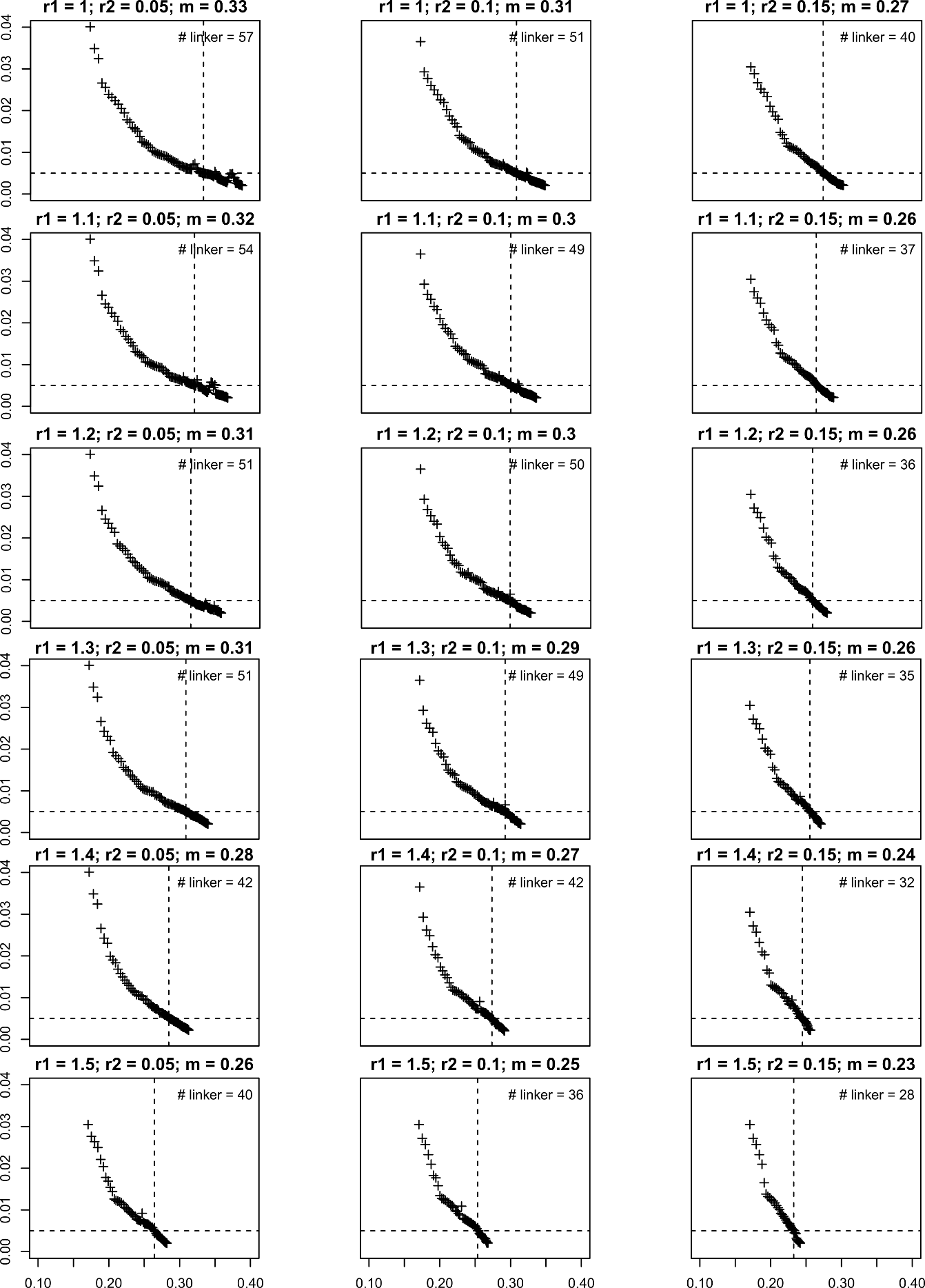


**Figure S5.** Threshold determination. X-axis: module score; y-axis: increase ratio. m: subnetwork score.


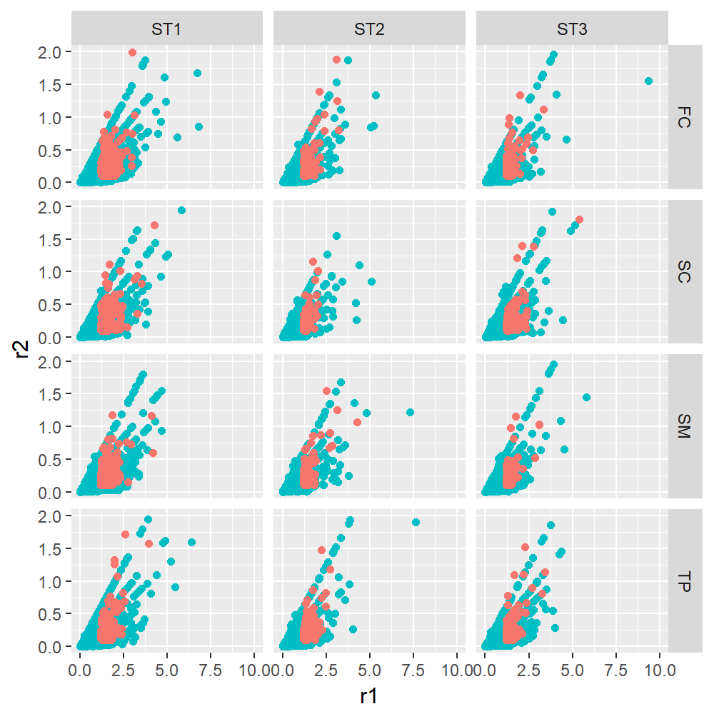

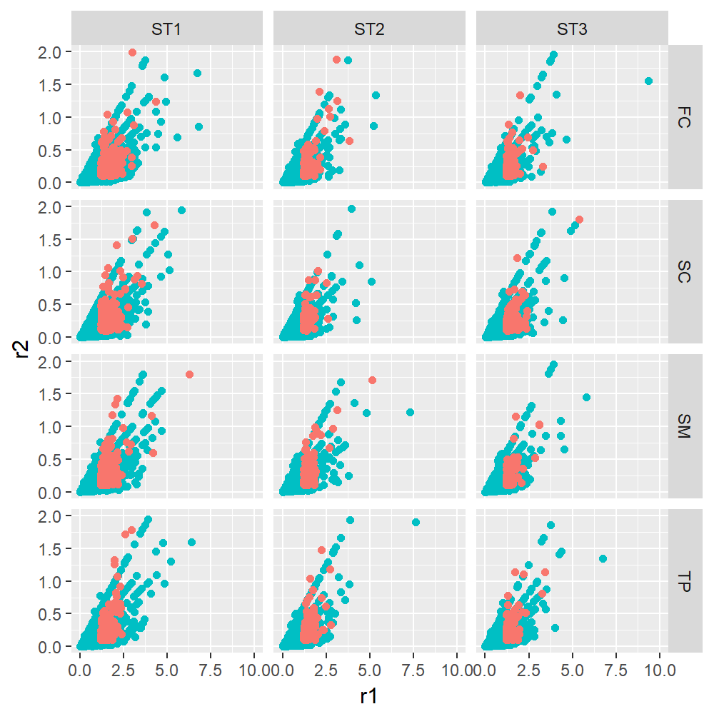


**Figure S6.** Distribution of r1 and r2. Each dot represents one node (gene) in the network that interacts with at least one seed gene. Red: nodes selected by r1 ≥ 1.2 and r2 ≥ 0.1, and interacting with ≥ 2 seed genes (the combination of CVgenes and DNMgenes). Four brain regions are labeled at the right of the panels. (A) Using the actual CVgenes or DNMgenes. (B) Using randomly selected seed genes with the same number of the actual CVgenes or DNMgenes.


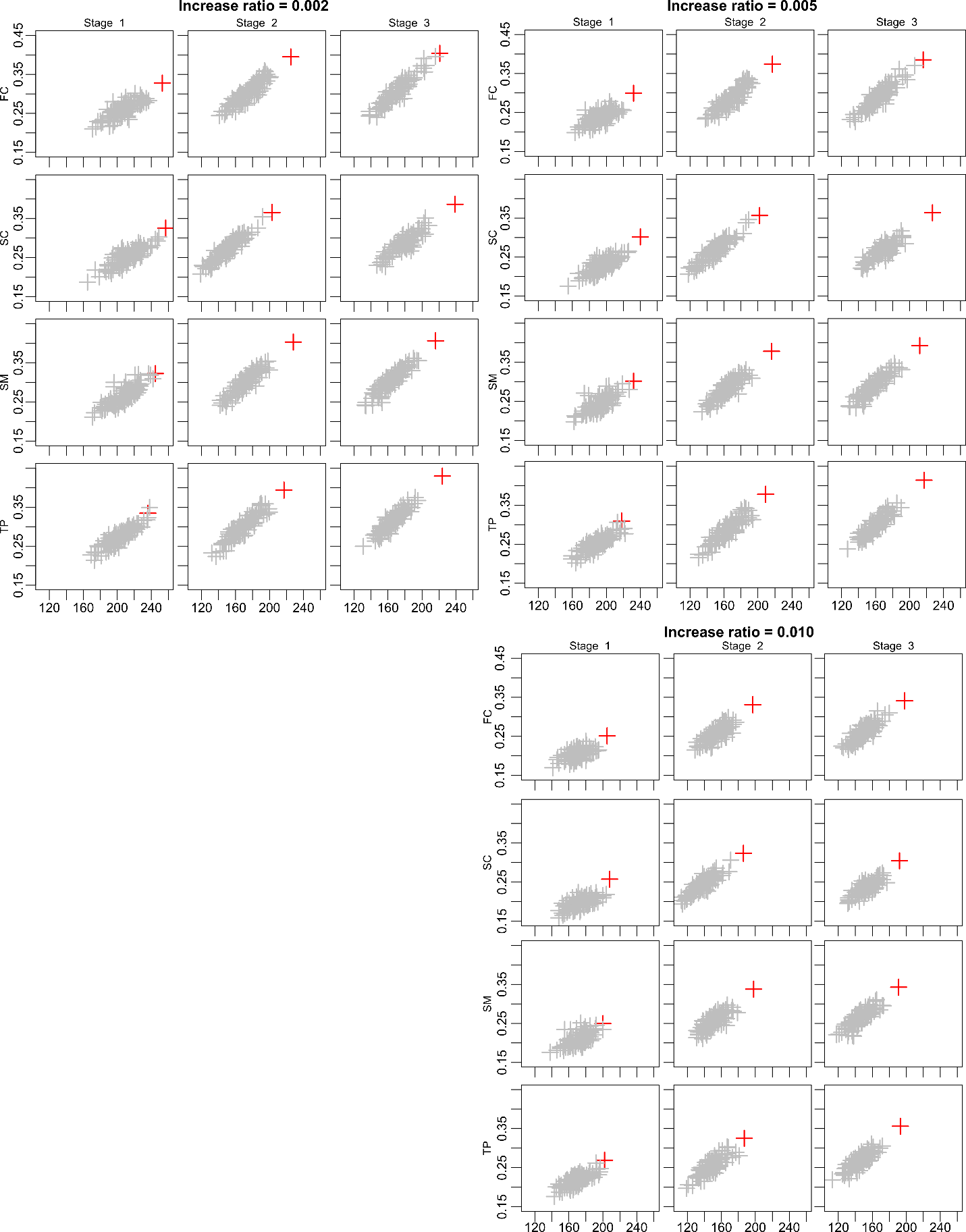


**Figure S7.** Comparison of subnetworks obtained at different threshold: 0.002, 0.005, and 0.01. X-axis: the number of genes of interest (i.e., the union of DNMgenes and CVgenes) in the stable subnetwork; y-axis: score of the subnetwork (see main text for details).

**
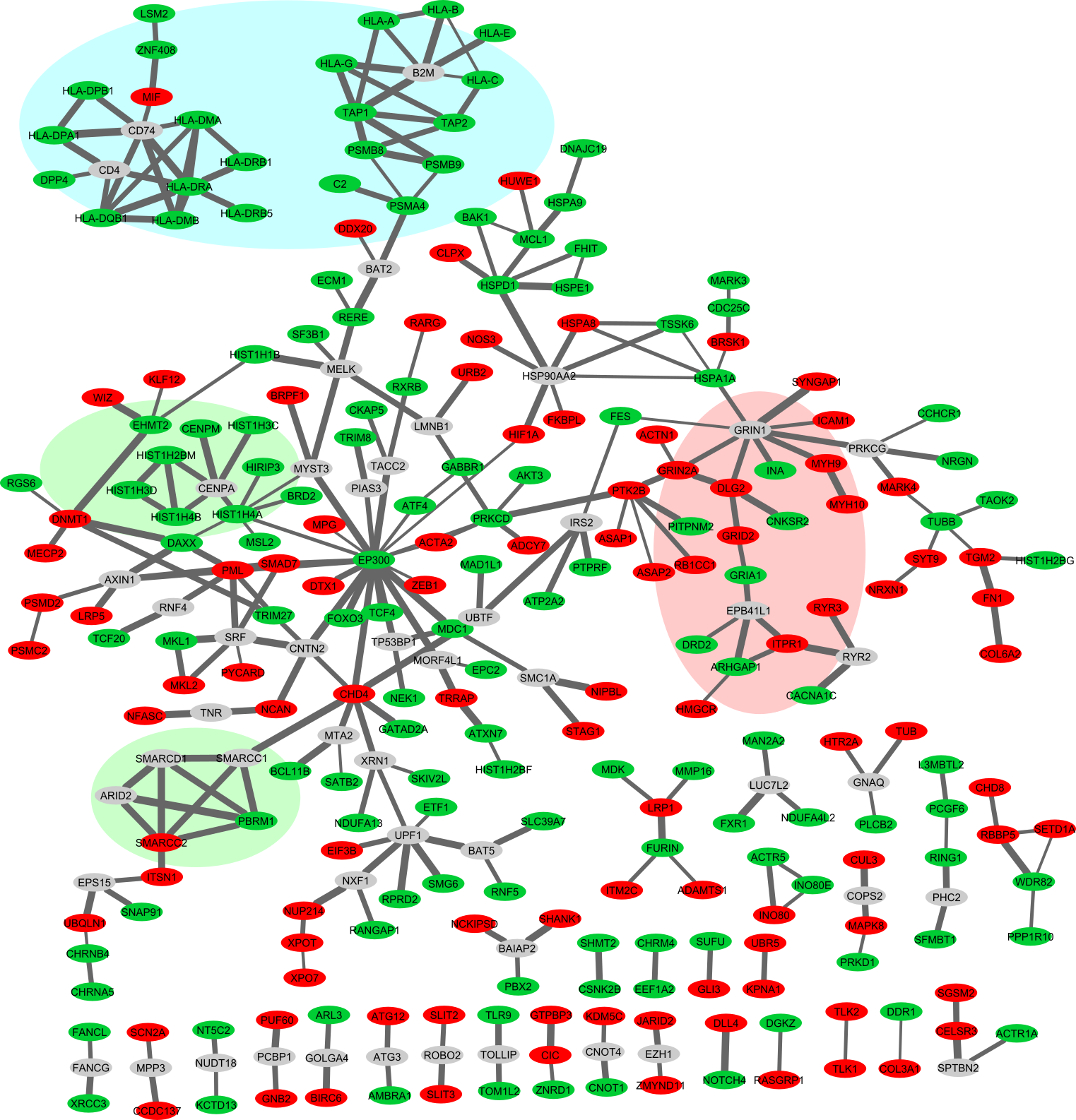
**

**Figure S8**. The schizophrenia subnetwork in FC, stage 1. Node color: red node: DNMgenes; green: CVgenes; and grey: link genes. Edge width is proportional to co-expression level measured by Pearson Correlation Coefficient (*PCC*) in FC, stage 1. Genes in chromatin remodeling pathway were highlighted in green area. Genes in MHC regions were highlighted in blue area. Genes in neurotransmitter activities were highlighted in red area.

**
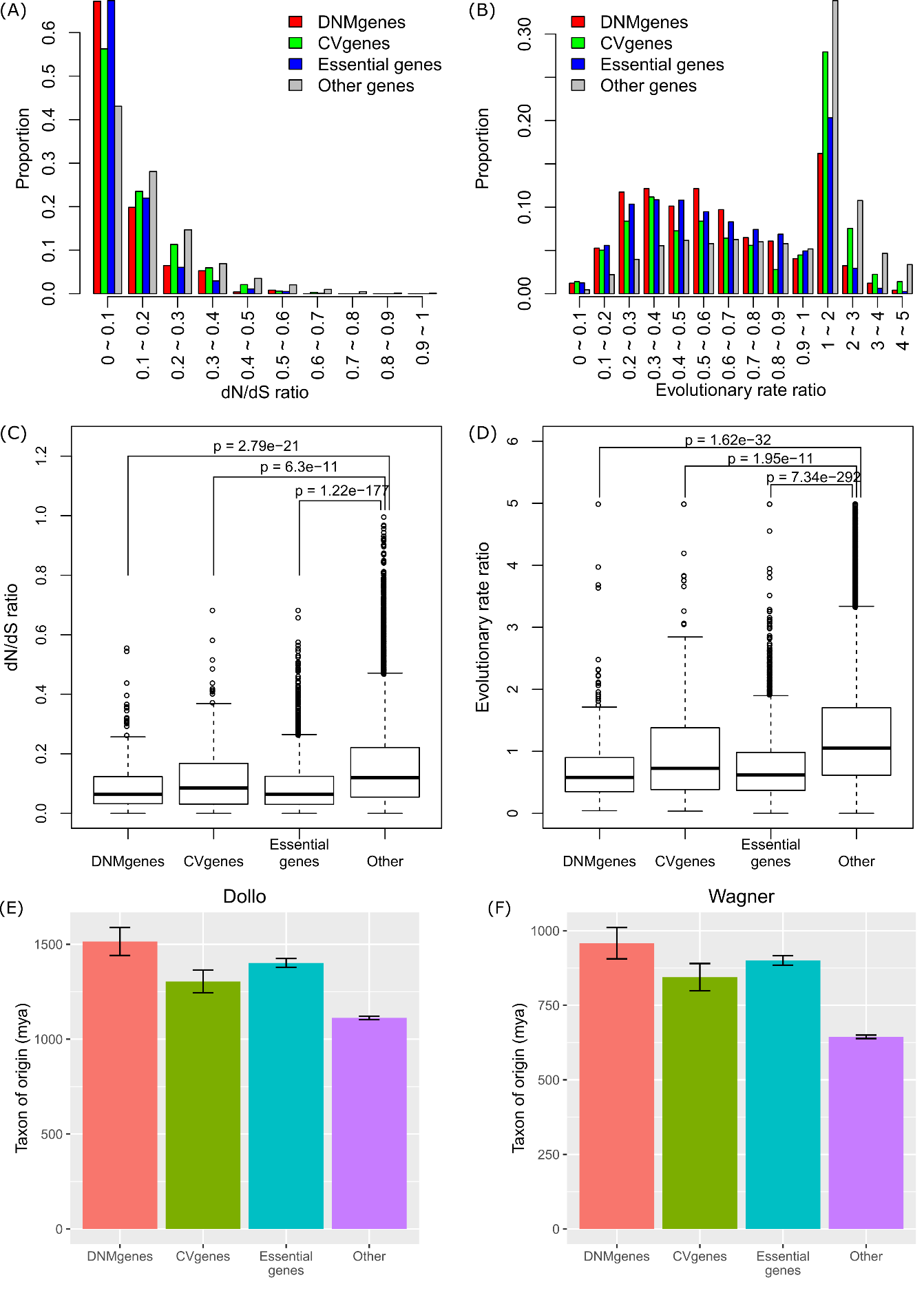
**

**Figure S9**. Evolutionary characteristics of four gene sets: DNMgenes, CVgenes, essential genes, and other genes (non-DNMgenes, non-CVgenes, and non-essential genes). Essential genes were downloaded from Georgi et al.,^1^ which reported 2472 human essential genes. (A) Distribution of the dN/dS ratio of the four gene sets. In the group with the smallest dN/dS ratio (0-0.1, indicating strong natural selection), a higher proportion of DNMgenes (67.21%), CVgenes (56.25%), and essential genes (67.37%) were observed, compared to other genes (43.08%). (B) Distribution of the evolutionary rate. The evolutionary rates of human proteins were calculated based on the eukaryotic orthologs from 103 genomes.^2, 3^ (C-D) Boxplot view of the distribution of dN/dS ratio (C) and evolutionary rate (D) for each gene set. The p-values were obtained using two-sample, unpaired t-test comparing a pair of gene groups, i.e., DNMgenes versus other genes, CVgenes versus other genes, and essential genes versus other genes, respectively. (E-F) Comparison of the average protein age (million years ago, mya) of each gene set estimated by the Dollo (E) and Wagner (F) parsimony method. Protein age was estimated using the ProteinHistorian method.^4^ We chose the results based on the PPODv4_PTHR7-OrthoMCL protein family database, with two sets of protein age estimated using the Dollo and Wagner parsimony, respectively.

**Table S1.** Description of BrainSpan expression data

|  | **Frontal cortex (FC)** | **Sub-cortical regions (SC)** | **Sensory-motor regions (SM)** | **Temporal-parietal cortex (TP)** |
| --- | --- | --- | --- | --- |
| **Fetal (13 ­to 26 postconception weeks, stage 1)** | FC-ST1 (n=42) | SC-ST1 (n=41) | SM-ST1 (n=37) | TP-ST1 (n=31) |
| **Early infancy to late childhood (4 months to 11 years, stage 2)** | FC-ST2 (n=45) | SC-ST2 (n=39) | SM-ST2 (n=44) | TP-ST2 (n=34) |
| **Adolescence to adulthood (13 ­to 23 years, stage 3)** | FC-ST3 (n=24) | SC-ST3 (n=20) | SM-ST3 (n=24) | TP-ST3 (n=18) |

**References**

1. Georgi B, Voight BF, Bucan M. From mouse to human: evolutionary genomics analysis of human orthologs of essential genes. *PLoS genetics* 2013; **9**(5)**:** e1003484.

2. Cheng F, Jia P, Wang Q, Lin CC, Li WH, Zhao Z. Studying tumorigenesis through network evolution and somatic mutational perturbations in the cancer interactome. *Mol Biol Evol* 2014; **31**(8)**:** 2156-2169.

3. Bezginov A, Clark GW, Charlebois RL, Dar VU, Tillier ER. Coevolution reveals a network of human proteins originating with multicellularity. *Mol Biol Evol* 2013; **30**(2)**:** 332-346.

4. Capra JA, Williams AG, Pollard KS. ProteinHistorian: tools for the comparative analysis of eukaryote protein origin. *PLoS computational biology* 2012; **8**(6)**:** e1002567.
